# Effects of Carbohydrate and Glutamine Supplementation on Oral Mucosa Immunity after Strenuous Exercise at High Altitude: A Double-Blind Randomized Trial

**DOI:** 10.3390/nu9070692

**Published:** 2017-07-03

**Authors:** Aline Venticinque Caris, Edgar Tavares Da Silva, Samile Amorim Dos Santos, Sergio Tufik, Ronaldo Vagner Thomatieli Dos Santos

**Affiliations:** 1 Department of Psychobiology, Universidade Federal de São Paulo, São Paulo 04032-020, Brazil; alinecaris@hotmail.com; 2 Department of Bioscience, Universidade Federal de São Paulo, Santos 11015-020, Brazil; edgartavares@uol.com.br (E.T.D.S.); samile.unifesp@gmail.com (S.A.D.S.); sergiotufik@zipmail.com.br (S.T.)

**Keywords:** supplementation, carbohydrate, hypoxia, physical exercise, glutamine, high altitude, innate immune response, oral mucosal immunity

## Abstract

This study analyzed the effects of carbohydrate and glutamine supplementation on salivary immunity after exercise at a simulated altitude of 4500 m. Fifteen volunteers performed exercise of 70% of VO_2peak_ until exhaustion and were divided into three groups: hypoxia placebo, hypoxia 8% maltodextrin (200 mL/20 min), and hypoxia after six days glutamine (20 g/day) and 8% maltodextrin (200 mL/20 min). All procedures were randomized and double-blind. Saliva was collected at rest (basal), before exercise (pre-exercise), immediately after exercise (post-exercise), and two hours after exercise. Analysis of Variance (ANOVA) for repeated measures and Tukey post hoc test were performed. Statistical significance was set at *p* < 0.05. SaO_2_% reduced when comparing baseline vs. pre-exercise, post-exercise, and after recovery for all three groups. There was also a reduction of SaO_2_% in pre-exercise vs. post-exercise for the hypoxia group and an increase was observed in pre-exercise vs. recovery for both supplementation groups, and between post-exercise and for the three groups studied. There was an increase of salivary flow in post-exercise vs. recovery in Hypoxia + Carbohydrate group. Immunoglobulin A (IgA) decreased from baseline vs. post-exercise for Hypoxia + Glutamine group. Interleukin 10 (IL-10) increased from post-exercise vs. after recovery in Hypoxia + Carbohydrate group. Reduction of tumor necrosis factor alpha (TNF-α) was observed from baseline vs. post-exercise and after recovery for the Hypoxia + Carbohydrate group; a lower concentration was observed in pre-exercise vs. post-exercise and recovery. TNF-α had a reduction from baseline vs. post-exercise for both supplementation groups, and a lower secretion between baseline vs. recovery, and pre-exercise vs. post-exercise for Hypoxia + Carbohydrate group. Five hours of hypoxia and exercise did not change IgA. Carbohydrates, with greater efficiency than glutamine, induced anti-inflammatory responses.

## 1. Introduction

Mucosal immunity, particularly in saliva, is considered the first line of defense against pathogens, because it contains numerous protective proteins. Some of these, such as salivary immunoglobulins (Igs), are involved in innate and adaptive immune responses [1]. In addition to Igs, there are also cytokines, such as interleukin (IL)-1ß, tumor necrosis factor (TNF)-α, and IL-6, that are used to assess the response to acute stress, stimulating immune cells, and modulating local inflammation [2,3].

Recent data suggest that exposure to hypoxia may modulate important aspects of innate immune responses [4], inflammation [5,6,7], and metabolism [8,9]. However, this issue has not been fully clarified, and only a few studies have been conducted under hypoxic conditions with the specific objective of investigating different immune/inflammatory parameters among humans [10].

It is known that exercise influences mucosal immunity, but the nature of this effect has not reached a consensus yet [11,12]. Some studies show that acute moderate-intensity exercise can result in a reduction of immunoglobulin A (IgA) concentration post-exercise; some do not describe any changes, while others report an increased concentration of IgA [13]. IgA is the most abundant protein in the antibacterial mucosal and it is considered the best indicator of oral mucosal immunity. Intense exercise causes a reduction in IgA levels [13] and increases inInterleukin-1 ß (IL-1ß), TNF-α, and IL-6 concentrations [2], resulting in poor performance of the immune function of the mucous membranes, increasing the incidence of upper respiratory tract infections (URTIs), and the emergence of other opportunistic diseases [14].

Thus, it is observed that exercise may modulate mucosal immunity under normal atmospheric pressure, but when exercise takes place in high altitude, it becomes a greater challenge for the body, since hypoxia and exercise are considered stressors that can act together. Evidence suggests that this combination may result in a more pronounced impact on the immune function of the oral mucosa and may trigger an intense immunosuppression [15,16].

On the other hand, studies have analyzed nutritional strategies that are efficient at sea level [17,18,19,20] to help mitigate the effects of exercise at altitude, providing better performance and prevention of infections [21].

When considering the anti-inflammatory effect of glutamine on stress factors, like exercise, harsh environments [21], and diseases (cancer, sepsis, burns, trauma) [22], this supplement can regress inflammation even during long-term exercises performed at sea level. Therefore, glutamine supplements have been shown to decrease the number of URTIs in athletes by promoting the production of IgA [23] and maintaining the balance of pro/anti-inflammatory markers [24].

On the other hand, carbohydrate supplements are used as a strategy to reduce the effects caused by the exercise on the immune system [25], and also contribute to improve performance. The intake of carbohydrate can significantly alter the immune response to intense exercise by attenuating the proliferation of lymphocytes, and by modulating cytokine pro/anti-inflammatory markers [26].

It has been shown that carbohydrate and glutamine supplements can be used isolated as a strategy to reverse the deteriorating mucosal immunity after strenuous exercise at sea level [23,27], however, the combined effect of both supplements is still not clarified.

In this context, we propose that the nutritional strategies used to prevent immune suppression after strenuous exercise at sea level can also be effective in hypoxic conditions. Thus, the objective of this study was to analyze the effect of carbohydrate and glutamine supplementation on oral mucosal immunity after exercise at a simulated altitude of 4500 m.

## 2. Methods

### 2.1. Experimental Design

This was a randomized, double-blind, placebo controlled crossover study and the sample size was determined using a statistics website from the Australian government [28]. After starting the clinical study, there were no changes in the methodology.

### 2.2. Participants

**Figure 1:**
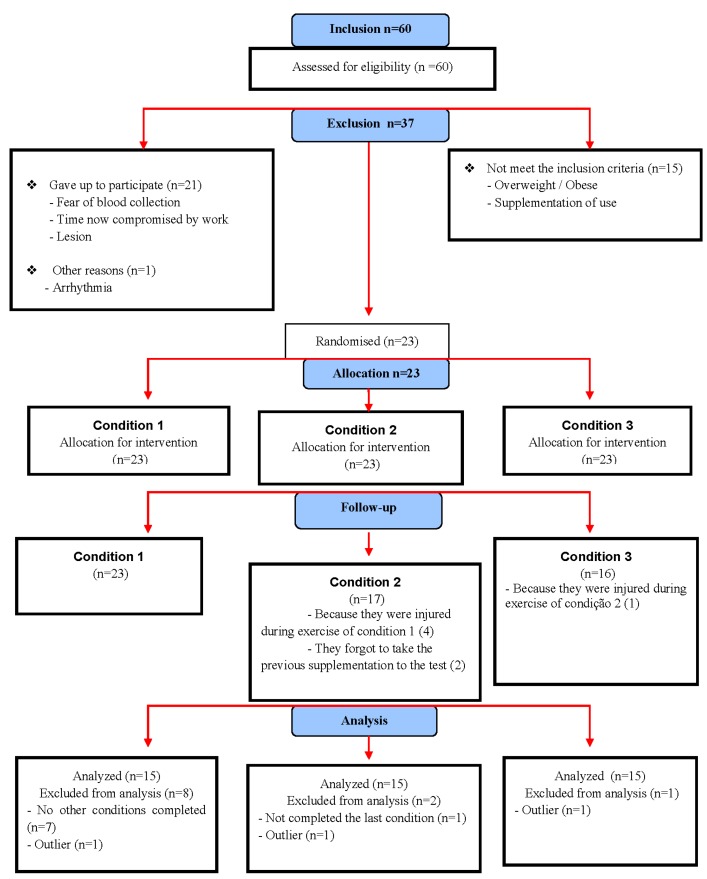
CONSORT flow diagram 2010.

The sample of this study included 15 healthy male volunteers (women were not included in the sample to avoid the possible influences of female sex hormones) that were physically active (performing physical activity at least 3x/week for 90 min each session) with the following physiological and anthropometric characteristics: age: 26.4 ± 3.9 years old; body mass: 73.7 ± 8.7 kg; height: 1.76 ± 0.02 m; Body Mass Index (BMI): 23.7 ± 2.5 kg/m^2^; VO_2peak_: 50.6 ± 5.4 mL/kg/min; maximum heart rate: 189.9 ± 8.2 beats per minute. Exclusion criteria were defined as: health problems; alterations in the electrocardiogram (ECG) at rest, stress and clinical evaluations, smoking, use of drugs, alcohol abuse, use of any medication that could interfere with the study results, and exposure to hypoxia during the previous six months. Figure 1 is a CONSORT flow diagram [29], explaining the stages of the randomized study. Initially, 60 volunteers were recruited to take part in the study, however 37 were eliminated based on the exclusion criteria. Of the 23 volunteers remaining, only 15 completed all the requirements.

Data were collected at the Interdisciplinary Laboratory for Exercise Physiology (LAIFE), Federal University of São Paulo (UNIFESP), São Paulo, between December 2014 and July 2015. The study procedures were approved by the Research Ethics Committee of the Federal University of São Paulo (Ethical approval code: 69 839/2014) on 4 March 2015 and are in accordance with the guidelines established by Resolution #466 of the Ministry of Health and the International Declaration of Helsinki.

### 2.3. Intervention

The participants came to the laboratory four times, with an interval of six days between each visit. During the first session, relevant information was presented, which consisted of objectives, procedures, guidelines for not taking supplements, and only low-intensity exercise. The participants were randomized into three groups [30] and were asked to sign a consent form. Next, they performed resting ECG, stress and cardiopulmonary exercise tests. The blinding process occurred in order to offer supplements and placebos that had the same characteristics of color, consistency, smell, taste, and presentation. An individual, oblivious of the study, was responsible for delivering the supplements to the participants every week, so the researches had no contact with the supplements. During the next three visits, the participants performed three random, blinded exercise sessions:

1. Group Hypoxia (Exercise + Altitude + Placebo): Participants consumed glutamine placebo supplements during the six days prior to the test (10 g corn starch + 10 g lactose), taken in the evening. During test day, they performed an exercise session at 70% of VO_2peak_ at a simulated altitude of 4500 m and were given a carbohydrate placebo supplement (Crystal Light^®^—Kraft Foods, Inc. strawberry, Chicago, IL, USA), 200 mL every 20 min during exercise and during recovery for two hours.
2. Group Hypoxia + CHO (Exercise + Altitude + Carbohydrate): Participants consumed glutamine placebo supplements during the six days prior to the test (10 g corn starch + 10 g lactose) taken in the evening. During test day, they performed an exercise session at 70% of VO_2peak_ at a simulated altitude of 4500 m, and were given carbohydrate supplements (Maltodextrin strawberry flavor—Probiótica^®^—Laboratories, Embu das Artes, São Paulo, Brazil), 200 mL at a concentration of 8% every 20 min during exercise and during recovery for two hours.
3. Group Hypoxia + GLN (Exercise + Altitude + Carbohydrate + Glutamine): Participants consumed 20 g of glutamine (Probiótica^®^—Laboratories, Embu das Artes, São Paulo, Brazil) in the six days prior to the test, between 8:00–10:00 p.m. During test day, they performed an exercise session at 70% of VO_2peak_ at a simulated altitude of 4500 m, and were given carbohydrate supplements (Maltodextrin strawberry flavor—Probiótica^®^—Laboratories, Embu das Artes, São Paulo, Brazil), 200 mL at a concentration of 8% every 20 minutes during exercise and during recovery for two hours.

For all exercise sessions, water intake was ad libitum. However, there was no control of the ingested volume.

#### Determination of VO_2peak_

To determine the VO_2peak_ in normoxic conditions, a test was performed with progressive intensity on a treadmill (LifeFitness^®^- 9700HR, Rosemont, IL, USA) with an initial speed of 7 km/h and increase of 1 km/h every minute until exhaustion (defined as the incapacity to keep up with the speed of the treadmill for 15 s or until the volunteer requested to stop the test after being encouraged to continue [31]) The encouragement for the volunteers was similar in all tests and carried out by the same person. During the test, we used a fixed inclination of 1% to simulate the physical stress of field tests [32].

Heart rate was monitored with a Polar Vantage NV watch (*Polar*^®^, Sark Products, Waltham, MA, USA), blood pressure was monitored by sphygmomanometer and stethoscope, and perceived exertion by the Borg scale (6 to 20) [33]. The respiratory parameters were measured by a gas analyzer (Cosmed Quark PFT model, Albano Laziale, Rome, Italy), pulmonary function (FRC & DLCO, Albano Laziale, Rome, Italy) was analyzed using a facemask (Hans Rudolph Inc., Shawnee, KS, USA). All calibration procedures were performed according to the manufacturer’s recommendations.

### 2.4. Altitude Simulation

A normobaric chamber was used (normobaric chamber CAT—Colorado Altitude Training™/CAT-12 Air Unit^®^, Lousiville, CO, USA) to simulate an altitude of 4500 m (changing carbon dioxide and oxygen concentrations (equivalent to a barometric pressure of 433 mmHg and a fraction of inspired oxygen of 13.5% O_2_)).

### 2.5. Sessions of Exercise and Recovery

The participants spent the first two hours in the hypoxic chamber at rest and then began to exercise on a treadmill (LifeFitness^®^- 9700HR, Rosemont, IL, USA) with a fixed inclination of 1% and intensity of 70% of VO_2peak_ until exhaustion or up to one hour. After exercising, they remained in the chamber for two more hours for recovery. Each test was followed by six days of rest, which was considered long enough to eliminate the effects of hypoxia [34] and supplementation [24]. All exercise session were performed after an overnight of fasting to avoid possible influences of diet and to maintain a standardized metabolic condition. Testing began at 7:30 a.m. to avoid circadian influences.

### 2.6. Hemoglobin O_2_ Saturation (SaO_2_%)

During all tests, the SaO_2_% was monitored by a pulse oximeter on the finger (FingerPulse^®^, MD300C202 model, Beijing, China) and assessed during four stages with saliva collection.

### 2.7. Saliva Collection

The saliva samples were collected using the Salivet method (cylindrical roller bearings that absorb saliva during the period of a minute) during four moments: immediately before entering the chamber (baseline), immediately before starting exercise (pre-exercise), immediately after exercise (post-exercise), and after two hours of recovery (after 2:00). After collection, the sample was put into a tube and centrifuged at a speed of 600× *g* for 20 min. Then, a clear fluid specimen was obtained and stored frozen (−80 °C) for analysis.

### 2.8. Determinants in Saliva

IgA was determined by immunoturbidimetric method using Kits from Labtest^®^ (Lagoa Santa, MG, Brazil) and cytokines (TNF-α, IL-6, and IL-10) were determined using Milliplex Kits^®^ (Darmstadt, Germany).

The flow rates of IgA, TNF-α, IL-6, and IL-10 were calculated by multiplying the concentration of each parameter by salivary flow (mL/min) as described by Usui et al. (2011) [2].

### 2.9. Statistical Analysis

Data normality was verified by the Shapiro-Wilk test. Descriptive analysis consisted of mean and standard error. ANOVA for repeated measures followed by post hoc Tukey test verified the interactions between groups and time, and Cohen’s d was calculated to estimate effect size: 0.20–0.30 = small effect size; 0.40–0.70 = medium effect size, and ≥0.80 = large effect size. The software Statistics^®^ 7.0 (StatSoft, Inc., Tulsa, OK, USA) was used for the statistical analyzes and the level of significance was set at *p* < 0.05.

## 3. Results

The results are presented in tables and figures. There was no significant difference between groups Hypoxia, Hypoxia + Carbohydrate, and Hypoxia + Glutamine for SaO_2_% (F = 2.2, *p* = 0.119), as shown in Table 1. However, significant differences were observed regarding time (F = 248.5, *p* < 0.001). When comparing the moment of measurement, we found reduction of SaO_2_% at baseline versus pre-exercise (*p* < 0.001) and baseline versus post-exercise (*p* < 0.001). Additionally, there was reduction at baseline in relation to after recovery for all three groups (*p* < 0.01). There was also a reduction of SaO_2_% in pre-exercise versus post-exercise (*p* < 0.001) for the hypoxia group. However, an increase was observed in pre-exercise compared to recovery for both groups with supplements (*p* < 0.001), and between post-exercise and recovery for all groups (*p* < 0.001). There was significant difference in the interaction between the groups and time (F = 5.5, *p* < 0.001), with an increase in Hypoxia group compared to Hypoxia + Glutamine group in recovery (*p* = 0.02).

**Figure 2:**
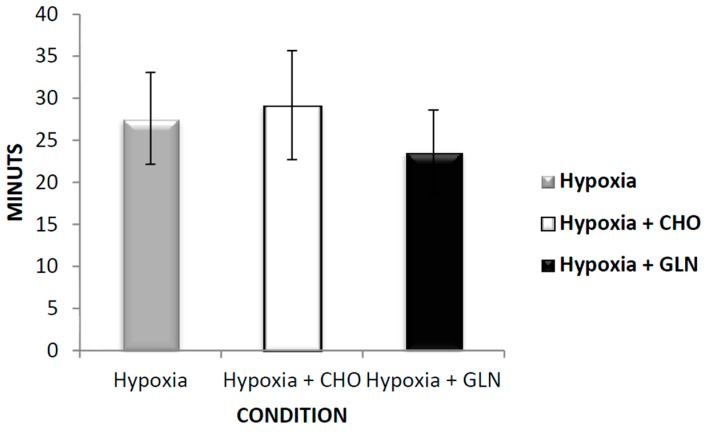
The results of time of exhaustion (min) was described by mean ± Standard Error (SE). The interaction of group versus time was analyzed by Analysis of Variance (ANOVA) for repeated measures followed by post hoc of Tukey test. The level of significance was set at *p* < 0.05. *n* = 15 volunteers. Hypoxia + CHO = Hypoxia + carbohydrate and hypoxia + GLN = hypoxia + glutamine.

Figure 2 shows results of time to exhaustion. There were no statistical differences regarding time to exhaustion between Hypoxia group (27.6 ± 5.46), Hypoxia + Carbohydrate group (29.2 ± 6.49), and Hypoxia + Glutamine group (23.66 ± 4.95).

Salivary flow (mL/min) results are presented in Table 2. No differences were observed between groups (F = 0.78, *p* = 0.925), but significant difference was found regarding time (F = 7.927, *p* < 0.001). There was an increase of salivary flow post-exercise versus recovery (*p* < 0.001) for the Hypoxia + Carbohydrate group. In the interaction of groups versus time, there was no significant difference (F = 0.863, *p* = 0.524).

**Figure 3:**
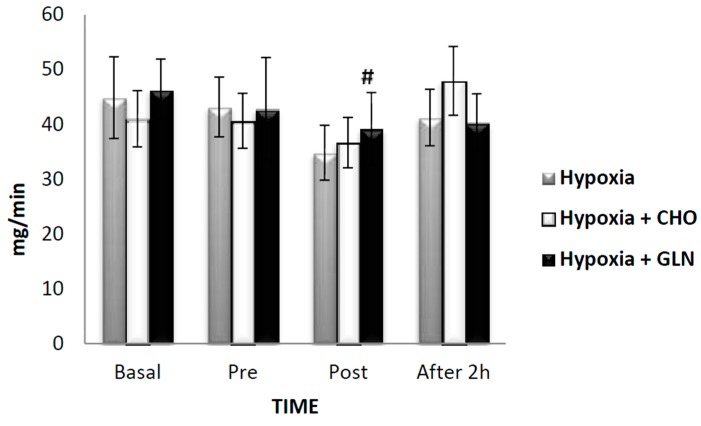
The results of secretory immunoglobulin A (IgA) (mg/min) was described by mean ± Standard Error (SE). The interaction of group versus time was analyzed by Analysis of Variance (ANOVA) for repeated measures followed by post hoc of Tukey test. The level of significance was set at *p* < 0.05. *n* = 15 volunteers. **#** Statistically significant in relation to basal. Hypoxia + CHO = Hypoxia + carbohydrate and hypoxia + GLN = hypoxia + glutamine.

Regarding immunity, we measured IgA and cytokines. The salivary concentration of IgA showed no difference between groups (F = 0.080, *p* = 0.923), time (F = 2.578, *p* = 0.057), and interaction (F = 1.133, *p* = 0.347). However, Cohen’s effect size d for the concentration of salivary IgA of pre-exercise versus post-exercise was 0.9 and pre-exercise versus recovery was 2.27 for the Hypoxia group; for the Hypoxia + Carbohydrate group, pre-exercise versus recovery showed effect size d = 1.06, and baseline versus post-exercise d = 1.9. The Hypoxia + Glutamine group showed effect size d = 2.6 for baseline versus recovery (Table 3). The Cohen’s effect size d > 0.08 is considered a high effect. IgA secretion rate presented no differences between groups (*F* = 0.074, *p* = 0.929). However, a significant difference was observed for time (F = 6.462, *p* < 0.001), and a reduction was found from baseline versus post-exercise (*p* < 0.001) for the group Hypoxia + Glutamine. The Hypoxia group showed a reduction of 22.5% post-exercise compared to baseline, with no statistical difference, but Cohen’s effect size d of 1.58. There was no interaction of groups versus time (*F* = 1.262, *p* = 0.280) (Figure 3).

Results related to pro- and anti-inflammatory cytokines are presented in Table 3. Regarding IL-10 concentration, there was no significant differences between groups (*F* = 0.148, *p* = 0.863), time (*F* = 0.893, *p* = 0.447), and interaction (*F* = 0.487, *p* = 0.817). Cohen’s effect size d for all comparisons related to IL-10 was below 0.2. This is considered a small effect size.

**Figure 4:**
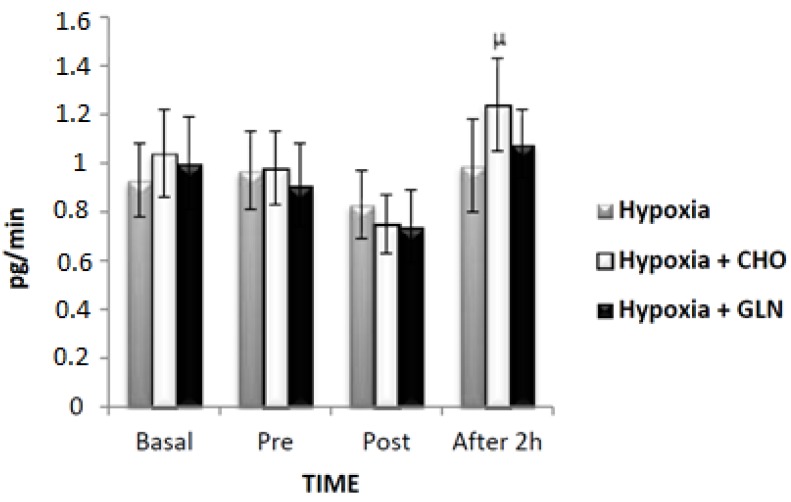
The results of interleukin (IL)-10 secretory (pg/min) was described by mean ± Standard Error (SE). The interaction of group versus time was analyzed by Analysis of Variance (ANOVA) for repeated measures followed by post hoc of Tukey test. The level of significance was set at *p* < 0.05. *n* = 15 volunteers. μ statistically significant in relation to post-exercise. Hypoxia + CHO = Hypoxia + carbohydrate and hypoxia + GLN = hypoxia + glutamine.

Regarding the rate of saliva secretion with IL-10 (Figure 4), there were no significant differences between the three groups (*F* = 0.170, *p* = 0.844), but there was a significant difference in time (*F* = 6.119, *p* < 0.001). Among these differences, there was an increase between post-exercise versus recovery (*p* < 0.001) for Hypoxia + Carbohydrate group. There was no interaction for this parameter (*F* = 0.589, *p* = 0.739).

The salivary concentrations of TNF-α (Table 3) showed no difference between the three groups (*F* = 0.48, *p* = 0.624). Regarding time, a reduction was observed (*F* = 15.88, *p* < 0.001) from baseline versus post-exercise (*p* = 0.001) and after recovery (*p* < 0.001) for the Hypoxia + Carbohydrate group. Similarly, a lower concentration was observed in pre-exercise time versus post-exercise (*p* < 0.001) and recovery (*p* < 0.001). There was no interaction of groups versus time (*F* = 1.61, *p* = 0.148).

**Figure 5:**
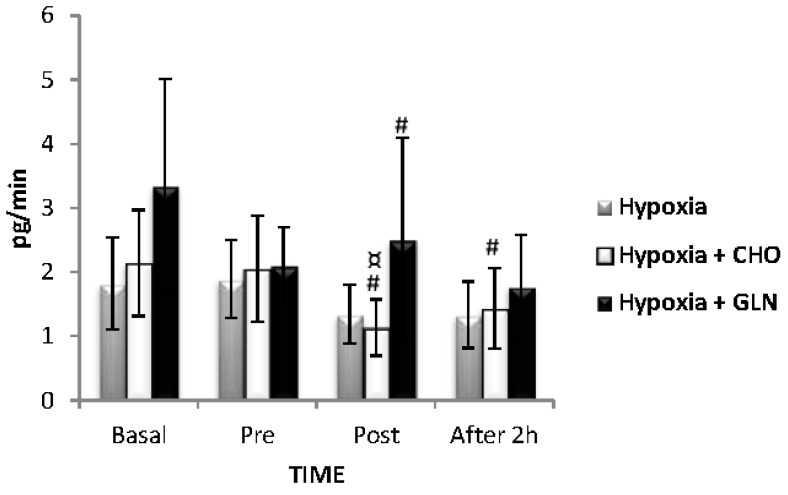
The results of tumor necrosis factor (TNF)-α secretory (pg/min) was described by mean ± Standard Error (SE). The interaction of group versus time was analyzed by Analysis of Variance (ANOVA) for repeated measures followed by post hoc of Tukey test. The level of significance was set at *p* < 0.05. *n* = 15 volunteers. **#** Different in relation to Basal. **¤** Statistically significant in relation to pre-exercise. Hypoxia + CHO = Hypoxia + carbohydrate and hypoxia + GLN = hypoxia + glutamine.

When considering the rate of saliva secretion of TNF-α (Figure 5), there was no significant difference between the three groups (*F* = 0.33, *p* = 0.723). However, there were differences in time for TNF-α secretion rate (*F* = 14.63, *p* < 0.001). Among these differences, we observed a reduction from baseline versus post-exercise (*p* < 0.001) for both supplemented groups, and a lower secretion of baseline compared to recovery (*p* < 0.001), and pre-exercise versus post-exercise (*p* < 0.001) for Hypoxia + Carbohydrate group. There was no interaction for this parameter (*F* = 3.01, *p* = 0.408).

**Figure 6:**
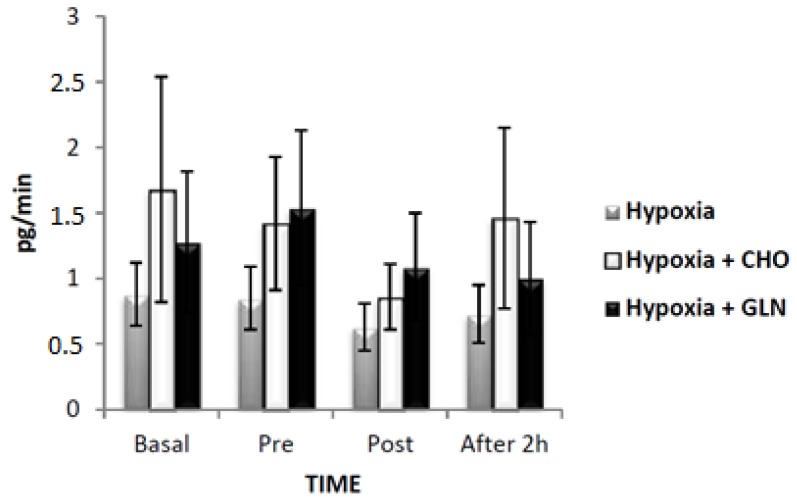
The results of IL-6 secretory (pg/min) was described by mean ± Standard Error (SE). The interaction of group versus time was analyzed by Analysis of Variance (ANOVA) for repeated measures followed by post hoc of Tukey test. The level of significance was set at *p* < 0.05. *n* = 15 volunteers. Hypoxia + CHO = Hypoxia + carbohydrate and hypoxia + GLN = hypoxia + glutamine.

Table 3 shows the salivary concentration of IL-6 and Figure 6 shows the salivary secretion rate of IL-6. Table 3 and Figure 4 showed no significant differences between groups (*F* = 0.213, *p* = 0.809) and (*F* = 0.138, *p* = 0.872), time (F = 3.200, *p* = 0.02) and (*F* = 2.555, *p* = 0.05), and interaction (F = 0.726, *p* = 0.629) and (*F* = 0.600, *p* = 0.730), respectively.

**Figure 7:**
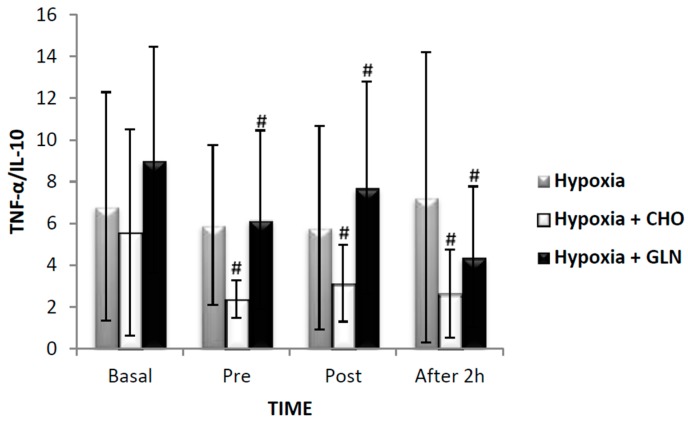
The results of salivary secretion rate of Tumor necrosis factor- α/Interleukin-10 (TNF-α/IL-10) was described by mean ± Standard Error (SE). The interaction of group versus time was analyzed by Analysis of Variance (ANOVA) for repeated measures followed by post hoc of Tukey test. The level of significance was set at *p* < 0.05. *n* = 15 volunteers. **#** Different in relation to Basal. Hypoxia + CHO = Hypoxia + carbohydrate and hypoxia + GLN = hypoxia + glutamine.

The ratio of salivary secretion rate of TNF-α/IL-10 is presented in Figure 7 and showed differences between groups (*F* = 5.2, *p* < 0.001), with an elevation of Hypoxia + Carbohydrate versus Hypoxia + Glutamine (*p* = 0.01). A significant difference was observed for time (*F* = 608.0, *p* < 0.001), and the reduction was found at baseline versus pre-exercise (*p* < 0.001), post-exercise (*p* < 0.001), and recovery (*p* < 0.001) in the group Hypoxia + Carbohydrate and Hypoxia + Glutamine. There was interaction of groups versus time (*F* = 4.6, *p* = 0.001), including an elevation between baseline in Hypoxia group compared to Hypoxia + Glutamine group (*p* = 0.01), and baseline in Hypoxia + Carbohydrate group compared to Hypoxia and Hypoxia + Glutamine group (*p* = 0.01).

## 4. Discussion

The aim of this study was to analyze the effect of carbohydrate and glutamine supplementation on oral mucosal immunity after exercise at a simulated altitude of 4500 m. The main finding of this study was that strenuous exercise associated with hypoxia, with or without supplementation, did not change salivary IgA. Despite the decrease in the pro/anti-inflammatory balance, an anti-inflammatory response was found in the group with carbohydrate supplementation because of changes in IL-10 and TNF-α concentrations.

According to several studies conducted at sea level, carbohydrate and/or glutamine supplementation have shown to be effective on mitigating the stress effects of vigorous exercise on the immune system. Taking into consideration that the number of people that travel to places of high altitudes for tourism, work, and sports increases each year, it becomes of great importance to elucidate the effects of carbohydrate and/or glutamine supplementation in hypoxic environments on the oral mucosal immunity, which is considered a practical method to indicate stress. Therefore, in the future, new interventions may be proposed and designed to minimize the effects of hypoxia among athletes, travelers, workers, and people chronically exposed to high altitudes.

Regarding results involving SaO_2_%, the values at baseline were not significantly different for the groups, since they are in normal oxygen concentration. However, a reduction in SaO_2_% was found after two hours of exposure for all groups, proving the efficiency of the hypoxia model used in this study and confirming the results found in the studies conducted by Tannheimer et al. [35], Mazzeo [14], and Pomidori et al. [36]. However, after two hours of recovery in hypoxia, SaO_2_% increased almost immediately post-exercise, suggesting recovery. However, this was not enough time to restore the values to baseline levels.

The SaO_2_% results of the Hypoxia + Carbohydrate group were similar to the group with no supplementation at baseline, but different after two hours of recovery with an increase in SaO_2_% compared to pre-exercise and post-exercise. Such modifications are related to carbohydrate intake, increasing the concentration of CO_2_ to a level that stimulates ventilation, thus enhancing blood oxygenation and reducing the desaturation of hypoxia [37,38]. The Hypoxia + Glutamine group showed similar changes compared to the Hypoxia group at baseline, although the results were different after two hours of recovery, showing an increase at pre-exercise, post-exercise, and hypoxic condition. The reasons for the restoration of SaO_2_% in the Hypoxia + Glutamine group are not known, but it is suggested that the increased availability of plasma glutamine may interfere with the central synthesis of glutamate, an excitatory neurotransmitter that stimulates ventilation [39], and thus contribute to SaO_2_% recovery.

Despite not evaluating plasma concentration of glutamine, a previous study with a similar protocol showed a 65.8% increase of glutamine post-exercise when compared to pre-exercise [24], reinforcing our hypothesis. Another fact to consider in the Hypoxia + Glutamine group is the combined action of the two supplements [40], contributing to increased ventilation in different pathways [37,39].

Pilardeau et al. [41] were the first to describe salivary flow in hypoxia; today it is known that salivary secretion can be affected by neural control of the autonomic nervous system, which indirectly regulates salivary flow and saliva composition [13]. The stress of intense exercise added to hypoxic environment stimulates the sympathetic nervous system, contracting blood vessels in salivary glands, which leads to a reduction in flow rate [27]. Our results are partly explained by these mechanisms, showing that salivary flow in hypoxic condition reduced 17% from baseline. However, the intake of both supplements appears to alleviate this effect, since the Hypoxia + Carbohydrate group showed a reduction of only 14% in salivary flow post-exercise compared to baseline. Interestingly, after two hours of recovery, supplementation with carbohydrates was able to promote a significant increase of 27% in flow compared to the end of the exercise. Bishop et al. [27] analyzed participants in normoxic conditions after two hours of riding a bicycle at 60% of VO_2max_ and found that consumption of carbohydrates (60 gL) increased salivary flow one hour after exercise, probably due to a reduction of sympathetic/parasympathetic balance [1].

The changes in salivary flow during and after exercise directly affect the concentration of salivary IgA [13,42], however, our results showed that the stimulation of salivary flow followed by the stress of exercise and hypoxia was not able to promote changes of total IgA concentration in any of the conditions. These results are similar to a study by Svendsen et al. [43] that analyzed participants exposed to hypobaric hypoxia conditions equivalent to 2000 m during 75 min of cycling at 70% VO_2peak_. This finding may have occurred due to the high intensity of exercise and its immunosuppressive function, preventing the elevation of IgA [44], or because the reduced time of exposure to hypoxia was not enough to modify secretion of IgA [1].

The IgA secretion rate in the Hypoxic group was reduced by 22.5% post-exercise compared to baseline; statistically this reduction was not significant, but it can be physiologically important, since IgA is the most abundant protection protein in saliva [1]. The lower level of IgA secretion indicates a specific reduction in the synthesis and/or secretion of salivary IgA in response to stress created by intense exercise [18,45] coupled with hypoxia. When supplements were taken by the participants, this reduction was slightly smaller (i.e., 10.6% in the Hypoxia + Carbohydrate group and 15.5% in the Hypoxia + Glutamine group). Our findings are similar to the study conducted by Krzywkowski et al. [45], involving normoxia with glutamine supplementation (17.5 g), which showed the same tendency of exercise (two hours of bicycle exercise at 75% VO_2max_) to reduce salivary IgA during and up to two hours after exercise.

The production of IgA in saliva may be mediated by several factors, such as stress hormones, nutritional factors, circadian cycle, hydration, alcohol intake, and also cytokines [1,2,13,46]. The effects of exercise on the production of cytokines in saliva are not well understood [2], especially in hypoxia [21]. Therefore, the present study was the first to investigate the effect of carbohydrate and glutamine supplementation on concentration of cytokines after exercise in hypoxic condition. 

The concentration of IL-10 and its secretion rates were not different when comparing time for any of the groups. However, despite the secretion rate being slightly lower in post-exercise when compared to baseline, we found that supplementation with carbohydrates was able to increase IL-10 after two hours of recovery, showing that the anti-inflammatory role of carbohydrate [46] can also be observed in saliva, thereby contributing to the maintenance of homeostasis at this site and helping to preserve mucosal immune responses [47]. We found similar results in the Hypoxia + Glutamine group, suggesting that, despite the increase of IL-10 by approximately 25% post-exercise compared to baseline, glutamine supplementation was not able to modulate the pro/anti-inflammatory balance by modification of IL-10.

The concentration and rate of secretion of TNF-α did not change in the Hypoxia group, however, in normoxic conditions, an increase of TNF-α was found in saliva during and after intense exercise [2], and a reduction after one-hour of recovery [48]. When assessing the Hypoxia + Carbohydrate group, TNF-α decreased after exercise and recovery, suggesting that the increase of IL-10 in saliva, mediated by supplementation with carbohydrates, may be responsible for a decrease in TNF-α secretion rate, similar to what occurs in other tissues, and enhancing the anti-inflammatory role of carbohydrates. In fact, supplementation with carbohydrates was able to attenuate the inflammatory process promoted by exercise and hypoxia, and modulated the balance between pro- and anti-inflammatory cytokines in saliva, as in normoxic conditions [49].

Regarding glutamine supplementation, we observed a decrease in TNF-α secretion rate immediately after exercise, and the reduction was more evident at the end of the second hour of recovery. These results demonstrated the anti-inflammatory role of glutamine in saliva, which has been observed in other tissues, directing the pro-inflammatory/anti-inflammatory balance toward an anti-inflammatory response [21,22].

The salivary concentration and secretion rate of IL-6 did not change in any of the groups. There are no results in the literature showing the effect of exercise on salivary IL-6 in hypoxia. Our results contradict the findings of Usui et al. [2], who observed an increase of IL-6 levels in saliva in normoxic conditions during and after exercise at 75% of VO_2max,_ including 80 min post-exercise. Thus, we cannot suggest what mechanisms are responsible for regulating IL-6 in hypoxia, but they are probably different from those in normoxic condition (i.e., maintaining homeostasis of blood and hepatic glucose and stimulating the release of C Reactive Protein (CRP). We believe this finding is a reflection of the increased use of IL-6 in its hematopoietic purpose [50,51] because it causes almost immediate reduction of SaO_2_% and deterioration of O_2_ due to hypoxia.

## 5. Conclusions

We conclude that five hours in hypoxia associated with strenuous exercise was not enough to promote a change in salivary IgA. Supplementation with carbohydrates and glutamine produced changes in the pro/anti-inflammatory balance, stimulating an inflammatory response in oral mucosa. However, our results should be interpreted with caution in regards to their generalizability because we only assessed male subjects.

## Figures and Tables

**Table 1:** O_2_ saturation percent (SaO_2_%).

		Condition		
		Hypoxia	Hypoxia + CHO	Hypoxia + GLN
SaO_2_%	Basal	97.13 ± 0.27	97.13 ± 0.21	96.87 ± 0.24
	Pre-exercise	85.47 ± 1.35 ^A^	82.40 ± 1.23 ^A^	84.33 ± 1.01 ^A^
	Post-exercise	79.67 ± 1.37 ^AB^	81.47 ± 1.02 ^A^	81.53 ± 1.43 ^A^
	2 h after	85.40 ± 1.00 ^AC^	89.33 ± 0.46 ^ABC^	90.27 ± 0.55 ^ABC^*

The results of SaO_2_ (%) were described by mean ± Standard Error (SE). The interactions of group versus time was Analysis of Variance (ANOVA) for repeated measures followed by Post hoc of Tukey test. The level of significance was set at *p* < 0.05. *n* = 15 volunteers. ^A^ statistically significant in relation to basal. ^B^ statistically significant in relation to pre-exercise. ^C^ statistically significant in relation to post-exercise. * Statistically significant in relation to hypoxia condition. Hypoxia + CHO = Hypoxia + carbohydrate and hypoxia + GLN = hypoxia + glutamine.

**Table 2:** Salivary Flow.

		Condition		
		Hypoxia	Hypoxia + CHO	Hypoxia + GLN
SaO_2_%	Basal	0.90 ± 0.11	0.89 ± 0.12	0.89 ± 0.09
	Pre-exercise	0.88 ± 0.11	0.81 ± 0.10	0.76 ± 0.08
	Post-exercise	0.75 ± 0.10	0.76 ± 0.11	0.79 ± 0.13
	2 h after	0.92 ± 0.11	1.04 ± 0.14 ^C^	0.85 ± 0.10

The results of Salivary Flow (mL/min) were described by mean ± Standard Errros (SE). The interactions of group versus time was Analays of Variance (ANOVA) for repeated measures followed by Post hoc of Tukey test. The level of significance was set at *p* < 0.05. *n* = 15 volunteers. ^C^ statistically significant in relation to post-exercise. Hypoxia + CHO = Hypoxia + carbohydrate and hypoxia + GLN = hypoxia + glutamine.

**Table 3:** IgA, IL-10, TNF-α e IL-6 Concentration

		Condition		
		Hypoxia	Hypoxia + CHO	Hypoxia + GLN
IgA	Basal	48.40 ± 2.07	46.47 ± 1.6	49.80 ± 2.27
	Pre-exercise	49.93 ± 2.21	50.47 ± 3.30	48.40 ± 1.67
	Post-exercise	47.33 ± 3.34	50.80 ± 3.88	45.60 ± 2.02
	2 h after	45.07 ± 1.25	46.73 ± 3.70	47.00 ± 2.60
IL-10	Basal	1.11 ± 0.15	1.22 ± 0.16	1.13 ± 0.17
	Pre-exercise	1.14 ± 0.17	1.27 ± 0.19	1.25 ± 0.20
	Post-exercise	1.08 ± 0.12	1.01 ± 0.13	1.02 ± 0.17
	2 h after	1.11 ± 0.16	1.22 ± 0.15	1.24 ± 0.15
TNF-α	Basal	2.06 ± 0.80	2.07 ± 0.69	3.69 ± 1.79
	Pre-exercise	2.18 ± 0.67	2.30 ± 0.80	2.74 ± 0.75
	Post-exercise	2.02 ± 0.98	1.54 ± 0.62 ^AB^	3.06 ± 2.25
	2 h after	1.34 ± 0.44	1.12 ± 0.39 ^AB^	2.03 ± 0.73
IL-6	Basal	1.05 ± 0.26	1.50 ± 0.68	1.42 ± 0.58
	Pre-exercise	0.93 ± 0.22	1.57 ± 0.51	1.92 ± 0.63
	Post-exercise	0.97 ± 0.30	1.15 ± 0.36	1.26 ± 0.48
	2 h after	0.82 ± 0.22	1.12 ± 0.37	1.06 ± 0.37

The results of concentration of Immunoglobulin A (IgA) (mg/dL), Interleukin-10 (IL-10), Tumoral Necrosis Factor-α (TNF-α) e Interleukin-6 (IL-6), in pg/mL were described by mean ± Standard Error (SE). The interactions of group versus time was Analaysis of Variance (ANOVA) *for repeated measures* followed by *Post hoc of Tukey* test. The level of significance was set at *p* < 0.05. *n* = 15 volunteers. ^A^ statistically significant in relation to basal. ^B^ statistically significant in relation to pre-exercise. Hypoxia + CHO = Hypoxia + carbohydrate and hypoxia + GLN = hypoxia + glutamine.

## References

[1] Born D.P., Faiss R., Willis S.J., Strahler J., Millet G.P., Holmberg H.C., Sperlich B. (2016). Circadian variation of salivary immunoglobin A, alpha-amylase activity and mood in response to repeated double-poling sprints in hypoxia. Eur. J. Appl. Physiol..

[2] Usui T., Yoshikawa T., Ueda S.-Y., Katsura Y., Orita K., Fujimoto S. (2012). Effects of acute prolonged strenuous exercise on the salivary stress markers and inflammatory cytokines. J. Phys. Fit. Sports Med..

[3] Slavish D.C., Graham-Engeland J.E., Smyth J.M., Engeland C.G. (2015). Salivary markers of inflammation in response to acute stress. Brain Behav. Immunity.

[4] Mishra K.P., Ganju L., Singh S.B. (2015). Hypoxia modulates innate immune factors: A review. Int. Immunopharmacol..

[5] Hartmann G., Tschöp M., Fischer R., Bidlingmaier C., Riepl R., Tschöp K., Hautmann H., Endres S., Toepfer M. (2000). High altitude increases circulating interleukin-6, interleukin-1 receptor antagonist and C-reactive protein. Cytokine.

[6] Hagobian T.A., Jacobs K.A., Subudhi A.W., Fattor J.A., Rock P.B., Muza S.R., Cymerman A., Friedlander A.L. (2006). Cytokine responses at high altitude: Effects of exercise and antioxidants at 4300 m. Med. Sci. Sports Exerc..

[7] Koeppen M., Eckle T., Eltzschig H.K. (2011). The hypoxia-inflammation link and potential drug targets. Curr. Opin. Anaesthesiol..

[8] Shay J.E., Celeste Simon M. (2012). Hypoxia-inducible factors: Crosstalk between inflammation and metabolism. Semin. Cell Dev. Biol..

[9] McNamee E.N., Korns Johnson D., Homann D., Clambey E.T. (2013). Hypoxia and hypoxia-inducible factors as regulators of T cell development, differentiation, and function. Immunol. Res..

[10] Mishra K.P., Ganju L. (2010). Influence of high altitude exposure on the immune system: A review. Immunol. Investig..

[11] Kunz H., Bishop N.C., Spielmann G., Pistillo M., Reed J., Ograjsek T., Park Y., Mehta S.K., Pierson D.L., Simpson R.J. (2015). Fitness level impacts salivary antimicrobial protein responses to a single bout of cycling exercise. Eur. J. Appl. Physiol..

[12] Rosa L., Teixeira A., Lira F., Tufik S., Mello M., Santos R. (2014). Moderate acute exercise (70% VO_2peak_) induces TGF-β, α-amylase and IgA in saliva during recovery. Oral Dis..

[13] Papacosta E., Nassis G.P. (2011). Saliva as a tool for monitoring steroid, peptide and immune markers in sport and exercise science. J. Sci. Med. Sport.

[14] Mazzeo R.S. (2005). Altitude, exercise and immune function. Exerc. Immunol. Rev..

[15] Shephard R.J. (1998). Immune changes induced by exercise in an adverse environment. Can. J. Physiol. Pharmacol..

[16] Mazzeo R.S. (2008). Physiological responses to exercise at altitude: An update. Sports Med..

[17] Castell L.M., Poortmans J.R., Newsholme E.A. (1996). Does glutamine have a role in reducing infections in athletes?. Eur. J. Appl. Physiol. Occup. Physiol..

[18] Castell L.M., Newsholme E.A. (1997). The effects of oral glutamine supplementation on athletes after prolonged, exhaustive exercise. Nutrition.

[19] Wong S.H., Williams C., Adams N. (2000). Effects of ingesting a large volume of carbohydrate electrolyte solution on rehydration during recovery and subsequent exercise capacity. Int. J. Sports Nutr..

[20] Nieman D.C., Henson D.A., Smith L.L., Utter A.C., Vinci D.M., Davis J.M., Kaminsky D.E., Shute M. (2001). Cytokine changes after a marathon race. J. Appl. Physiol..

[21] Walsh N.P., Gleeson M., Pyne D.B., Nieman D.C., Dhabhar F.S., Shephard R.J., Oliver S.J., Bermon S., Kajeniene A. (2011). Position statement. Part two: Maintaining immune health. Exerc. Immunol. Rev..

[22] Bailey N., Clark M., Nordlund M., Shelton M., Farver K. (2012). New paradigm in nutrition support: Using evidence to drive practice. Crit. Care Nurs. Q..

[23] Krieger J.W., Crowe M., Blank S.E. (2004). Chronic glutamine supplementation increases nasal but not salivary IgA during 9 days of interval training. J. Appl. Physiol..

[24] Caris A.V., Lira F.S., de Mello M.T., Oyama L.M., dos Santos R.V. (2014). Carbohydrate and glutamine supplementation modulates the Th1/Th2 balance after exercise performed at a simulated altitude of 4500 m. Nutrition.

[25] Nieman D.C. (2008). Immunonutrition support for athletes. J. Nutr. Rev..

[26] Carlson L.A., Kenefick R.W., Koch A.J. (2013). Influence of carbohydrate ingestion on salivary immunoglobulin A following resistance exercise. J. Int. Soc. Sports Nutr..

[27] Bishop N.C., Blannin A.K., Armstrong E., Rickman M., Gleeson M. (2000). Carbohydrate and fluid intake affect the saliva flow rate and IgA response to cycling. Med. Sci. Sports Exerc..

[28] National Statistical Service Sample Size Calculator.Disponivel em. http://www.nss.gov.au/nss/home.nsf/pages/Sample+Size+Calculator+Description?OpenDocument.

[29] Moher D., Hopewell S., Schulz K.F., Montori V., Gøtzsche P.C., Devereaux P.J. (2012). CONSORT 2010 explanation and elaboration: Updated guidelines for reporting parallel group randomised trials. Int. J. Surg..

[30] Research Randomizer. Disponível em. https://www.randomizer.org/.

[31] Sassi A., Marcora S.M., Rampinini E., Mognoni P., Impellizzeri F.M. (2006). Prediction of time to exhaustion from blood lactate response during submaximal exercise in competitive cyclists. Eur. J. Appl. Physiol..

[32] Jones A.M., Doust J.H. (1996). A 1% treadmill grade most accurately reflects the energetic cost of outdoor running. J. Sports Sci..

[33] Borg G.A. (1982). Psychophysical bases of perceived exertion. Med. Sci. Sports Exerc..

[34] Coppel J., Hennis P., Gilbert-Kawai E., Grocott M.P. (2015). The physiological effects of hypobaric hypoxia versus normobaric hypoxia: A systematic review of crossover trials. Extreme Physiol. Med..

[35] Tannheimer M., Thomas A., Gerngross H. (2002). Oxygen saturation course and altitude symptomatology during an expedition to broad peak (8047 m). Int. J. Sports Med..

[36] Pomidori L., Bonardi D., Campigotto F., Fasano V., Gennari A., Valli G., Palange P., Cogo A. (2009). The hypoxic profile during trekking to the Pyramid Laboratory. High Alt. Med. Biol..

[37] Golja P., Flander P., Klemenc M., Maver J., Princi T. (2008). Carbohydrate ingestion improves oxygen delivery in acute hypoxia. High Alt. Med. Biol..

[38] Charlot K., Pichon A., Richalet J.P., Chapelot D. (2013). Effects of a high-carbohydrate versus high-protein meal on acute responses to hypoxia at rest and exercise. Eur. J. Appl. Physiol..

[39] Honda Y., Tani H., Masuda A., Kobayashi T., Nishino T., Kimura H., Masuyama S., Kuriyama T. (1996). Effect of prior O_2_ breathing on ventilatory response to sustained isocapnic hypoxia in adult humans. J. Appl. Physiol..

[40] Favano A., Santos-Silva P.R., Nakano E.Y., Pedrinelli A., Hernandez A.J., Greve J.M. (2008). Peptide glutamine supplementation for tolerance of intermittent exercise in soccer players. Clinics.

[41] Pilardeau P., Richalet J.P., Bouissou P., Vaysse J., Larmignat P., Boom A. (1990). Saliva flow and composition in humans exposed to acute altitude hypoxia. Eur. J. Appl. Physiol. Occup. Physiol..

[42] Allgrove J.E., Gomes E., Hough J., Gleeson M. (2008). Effects of exercise intensity on salivary antimicrobial proteins and markers of stress in active men. J. Sports Sci..

[43] Svendsen I.S., Hem E., Gleeson M. (2016). Effect of acute exercise and hypoxia on markers of systemic and mucosal immunity. Eur. J. Appl. Physiol..

[44] Bishop N.C., Gleeson M. (2009). Acute and chronic effects of exercise on markers of mucosal immunity. Front. Biosci..

[45] Krzywkowski K., Petersen E.W., Ostrowski K., Link-Amster H., Boza J., Halkjaer-Kristensen J., Klarlund Pedersen B. (2001). Effect of glutamine and protein supplementation on exercise-induced decreases in salivary IgA. J. Appl. Physiol..

[46] Silva R.P., Natali A.J., Paula S.O., Locatelli J., Marins J.C.B. (2009). Imunoglobulina A salivar (IgA-s) e exercício: Relevância do controle em atletas e implicações metodológicas. Rev. Bras. Med. Esporte.

[47] Yamaoka M., Yamaguchi S., Okuyama M., Tomoike H. (1999). Anti-inflammatory cytokine profile in human heart failure: Behavior of interleukin-10 in association with tumor necrosis factor-alpha. Jpn. Circ. J..

[48] Rahman Z.A., Abdullah N., Singh R., Sosroseno W. (2010). Effect of acute exercise on the levels of salivary cortisol, tumor necrosis factor-alpha and nitric oxide. J. Oral Sci..

[49] Bishop N.C., Blannin A.K., Robson P.J., Walsh N.P., Gleeson M. (1999). The effects of carbohydrate supplementation on immune responses to a soccer-specific exercise protocol. J. Sports Sci..

[50] Faquin W.C., Schneider T.J., Goldberg M.A. (1992). Effect of inflammatory cytokines on hypoxia-induced erythropoietin production. Blood.

[51] Klausen T., Olsen N.V., Poulsen T.D., Richalet J.P., Pedersen B.K. (1997). Hypoxemia increases serum interleukin-6 in humans. Eur. J. Appl. Physiol. Occup. Physiol..

